# Handlers’ Expectations and Perceived Compatibility regarding the Partnership with Their First Guide Dogs

**DOI:** 10.3390/ani11102765

**Published:** 2021-09-22

**Authors:** Janice Lloyd, Claire Budge, Kevin Stafford

**Affiliations:** 1 College of Public Health, Medical and Veterinary Sciences, James Cook University, Townsville, QLD 4811, Australia; 2 THINK Hauora, 200 Broadway Avenue, Palmerston North 4410, New Zealand; clairebudge@gmail.com; 3 School of Agriculture and Environment, Massey University, Palmerston North 4474, New Zealand; k.j.Stafford@massey.ac.nz

**Keywords:** guide dogs, attachment, compatibility, expectations, guide dog matching, guide dog partnership, human-animal bond

## Abstract

**Simple Summary:**

Guide dogs assist people who are blind or visually impaired by primarily functioning as mobility aids, but also as companions and facilitators of social interactions. The level of success of the partnership depends on factors relating to work (mobility) as well as non-working (social) aspects of the relationship. This study examined how compatible 50 people (handlers) were with their first guide dogs, and how well the handlers’ expectations regarding mobility and social factors related to guide dog usage were met to assess the outcome of the partnership. Results indicate that dogs are meeting or exceeding the handlers’ expectations. Ratings of compatibility were high, especially concerning the emotional compatibility between handler and dog. However, the quality of the working relationship defined whether people considered the partnership to be successful or not. A tool to measure compatibility (accommodating both positive and negative aspects of the relationship) would be beneficial when matching a person with a suitable dog and to maximize the success of the guide dog partnership.

**Abstract:**

The main function of a guide dog is as a mobility aid, but they can also fulfill psychosocial roles as companions, social facilitators, and objects/providers of affection. This study examined the outcome of 50 peoples’ (handlers) partnerships with their first guide dog. Overall compatibility and the fulfilment of the handlers’ expectations regarding mobility and social factors related to guide dog usage were measured, and relationships between putative risk factors and the outcome of matching success were identified. The findings demonstrate that the dogs are generally exceeding expectations. The high average ratings of compatibility were notable, particularly with respect to the emotional compatibility between handler and dog. Comparing responses of those who felt the handler-dog pairing was a good match with those who felt it was a mismatch revealed it was the working aspects of the relationship that differentiated the two groups. However, the many aspects of life with a guide dog, beyond the complexities of the working relationship, suggest that a more nuanced compatibility measure accommodating both positive and negative aspects of the relationship could assist with matching, training and follow up of the handler-dog team to maximize success.

## 1. Introduction

The range of processes for selecting, breeding, raising, socializing and training suitable dogs to be mobility aids for people who are blind or visually impaired are complex, costly and time consuming [1]. The building of a successful guide dog team, comprising the user (handler) and dog, involves matching a suitable dog to the handler, training the handler and dog as a dyad and providing ongoing follow-up and support. In New Zealand (NZ) these roles are adopted by Blind Low Vision NZ Guide Dogs, which was formerly, and at the time of this study, known as the Royal New Zealand Foundation of the Blind (RNZFB) Guide Dog Services (GDS). This organization is the only one of its kind in NZ and services the entire country. Most guide dogs bred in NZ are Labrador Retrievers, Golden Retrievers, and purpose-bred crosses [2] plus a small number of Standard Poodles. Around 100 potential guide dogs are bred each year. Training takes approximately two years and costs upwards of NZD 50,000. At any one time, around 150 people in NZ are using a guide dog, and about another 50 are on the waiting list for one (M. Dawson, Breeding and Production Advisor, Blind Low Vision NZ, personal communication, 21 May 2021).

Guide dogs fulfill a variety of roles for their handlers. The dogs’ main function is as a mobility aid, enabling people to increase their mobility by locating and negotiating their way around obstacles, finding destinations, and maintaining independence [3,4,5]. Discussions with guide dog handlers suggest that getting around is less tiring with a dog than with a long cane as less effort is required [4,6]. However, as with companion/pet dogs, guide dogs can also fulfill other psychosocial roles as companions, objects and providers of physical contact and affection, and social facilitators [7,8,9]. Whitmarsh [5] interviewed over 400 guide dog handlers and found that not only did they feel more confident and independent as a result of having a guide dog, but they also experienced better social relationships and appreciated the companionship afforded by the dog. Some cited security and increased exercise as additional benefits [5]. Increased confidence has also been noted in other studies [8,10,11]. As illustrated above, prior to getting a guide dog, a person with a vision impairment is likely to have certain expectations around what a guide dog will provide them with. According to Refson et al. [12] these primarily relate to independence and mobility, but also include wanting more exercise, increased security and not wanting to use a long cane [5]. Guide dogs may even have a positive impact on health [12], although this may depend on the compatibility of the relationship [13].

Although more advantages than disadvantages have been noted in the literature concerning the use of guide dogs [7,8] and other assistance/service dogs [14], challenges that hamper these benefits do exist, and not all matches are equally successful [15]. The looks, behavior, health and temperament of the dog, and the physical and mental capabilities, personality and expectations of the potential handler may all influence the compatibility of the pairing and contribute to its success. In his exploration of the ways in which guide dog/handler partnerships were established in The Seeing Eye guide dog school of the 1930s, Pemberton described the “multispecies matchmaking” involved. He observed that “before arriving at the campus and meeting any dogs, the prospective guide dog owner had been matched with a canine partner by the instructors, in accordance with the individual physical capacities, temperaments, personalities and needs of each party” ([16], p. 98). While the term ‘compatibility’ is not applied to this process, there is clearly scope for compatibility, or the lack of it, to be applied to any of these matchmaking elements. The notion of compatibility between companion dogs and cats and their owners was explored by Budge et al. who defined it as the behavioral, physical and psychological fit between a companion animal and its owner [17] or, in this case, a guide dog and its handler.

Attachment or the emotional bond is another component of the relationship between humans and companion animals. This human-animal bond has been likened to that between a caregiver and a human infant [18], and more recently it has been suggested that dog-human attachment is more akin to adult pair bonds or friendships [19].The emotional bond is perhaps especially complex when the animal in question has a specific working, as well as social, role to play. Feelings of emotional attachment to the dog and perceptions of the bond is of major significance for the wellbeing of both handler and dog as it is likely to influence the success of the match [20].

In this paper the relationships between 50 guide dog handlers and their first dogs are considered. There were four goals:

- Goal 1: to explore the extent to which guide dog handlers considered their expectations to have been fulfilled by their first guide dog;
- Goal 2: to explore how compatible they considered the pairing of dog and handler to be with respect to both working (mobility) and non-working (social) aspects of the relationship;
- Goal 3: to see whether there were differences in the fulfilment of expectations and perceived compatibility between those who considered the handler/dog combination to be a matching success and those who considered it to be unsuccessful and judged it a mismatch;
- Goal 4: to examine whether the degree of motivation to (a) get a guide dog and (b) make a success of the relationship was associated with fulfilment of expectations, compatibility and matching success.

## 2. Materials and Methods

### 2.1. Study Participants

This study was part of a larger project, with further detail on recruitment, materials and methods being reported elsewhere [4,21]. In brief, all 240 current and previous members of the RNZFB GDS were invited to participate. A GDS staff member sent out the information packages and invitations in order to maintain confidentiality. A response rate of 71.9% was achieved (*N* = 151), with 50 of these volunteers being randomly selected to participate. They were interviewed in person (22%) or by phone (78%). Men constituted 48% of the sample, and ages ranged from 17 to 73 years at the time of first dog use (*M* = 37.6 years).

### 2.2. Instruments

To address the aims of this study, as described earlier, we focused on a subset of the questions asked of the guide dog handlers. No standardized measures of the variables in question were identified as being suitable for use with guide dog handlers and consequently a questionnaire was custom built.

#### 2.2.1. Fulfillment of Expectations

Fulfillment of expectations was measured with a set of five questions by asking “at the time you used this dog, were your expectations regarding …” in relation to (1) mobility (travel), (2) social function, (3) companionship, (4) dog’s behavior and (5) dog’s health. Each of these questions was rated on a 4-point Likert type scale ranging from expectations ‘not being met’ (rated as 1) through ‘somewhat met’ (rated as 2), ‘met’ (rated as 3) and expectations being ‘exceeded’ (rated as 4). Responses to these five items were combined to make a Fulfilment of Expectations Scale (FES).

#### 2.2.2. Compatibility

Compatibility was measured with a set of 10 items each rated on a 10-point rating scale representing ‘not at all compatible’ (rated as 1) to ‘extremely compatible’ (rated as 10). The content of the items and the associated variable names are presented in Table 1.

#### 2.2.3. Matching Success

The success of the match was measured as a dichotomous variable (success or not) by asking participants whether or not they considered the match between themselves and the dog to be a success.

#### 2.2.4. Motivation to Get and Succeed with a Dog

The two motivation questions were both rated on the same 10-point rating scale as the compatibility items. Participants were asked to rate (1) how motivated they were to acquire this dog (not at all—rated as 1, to extremely motivated—rated as 10) and (2) how motivated they were to succeed with this match (not at all—rated as 1, to extremely motivated—rated as 10).

### 2.3. Data Analysis

The data was analyzed using the statistical software package IBM SPSS Statistics 25. This work was exploratory and based on a small sample of non-normally distributed data, hence a descriptive rather than an inferential approach to data analysis was taken.

### 2.4. Ethical Consideration

Ethical approval was granted by Massey University NZ (ref. 98/204) and by all regions of the National Health Funding Authorities Human Ethics Committees (ref. 39/98 & 99/04) as participants were located around the country.

## 3. Results

### 3.1. Fulfilment of Expectations

The first goal was to examine how well participants’ expectations of having a guide dog were fulfilled by actually having and using the dog. The distribution of responses to the five fulfilment of expectations questions appears in Table 2, expressed as percentages, along with the results of a chi-square goodness of fit based on equal expected frequencies.

The most frequent response was that the dogs exceeded their handlers’ expectations in all respects; with companionship being rated highest, followed by social function and then mobility (travel). Expectations were met or exceeded for at least 72% and up to 98% of the handlers; the dog’s behavior being the lowest and companionship the highest. The chi-square results demonstrate that for all five variables, the observed frequencies were significantly different from the expected, with dogs meeting or exceeding expectations more often than not. As these variables were not normally distributed, most being negatively skewed, Spearman’s correlations were used to investigate how there were correlated (Table 3). These items were predominantly moderately positively correlated; the strongest correlation was between travel and behavior, the two work-related variables, the next strongest between travel and social function.

The five item scores were averaged to create the FES scale with a mean score of 3.38 (*SD* = 0.56).

### 3.2. Compatibility

The second goal was to examine how compatible participants considered the pairing of dog and handler to be with respect to the working and non-working related aspects of the relationship. As the data set was small and data were not normally distributed, the median responses on the 1–10 rating scale are presented in Table 4 along with the score ranges.

The median scores show that all 10 items were rated highly on average. However, the ranges revealed that there were some lower scores, particularly in relation to control, mobility, safety, and match. Due to the small sample size and non-normality of the data structure, Spearman’s rank correlations were used to examine the relationships between the compatibility items scores (Table 5).

The strongest correlation was found between satisfaction with the dog and with the match between handler and dog, followed by that between the bond and attachment variables. Strong correlations were also seen between the mobility, safety and control variables, and between bond and companionship. The pattern of correlations and the qualitative content of the items suggested they could contain two different subsets: one concerning the working ability of the dog as a useful, controllable and safe mobility aid, the other concerning the emotional or non-work-related variables of bonding, attachment and companionship. Consequently, items 3–5 and 8 were averaged to make a ’Work Compatibility’ subscale (*M* = 8.52, *SD* = 1.72) and items 1, 2 and 10 were averaged to create an ‘Emotional Compatibility’ subscale (*M* = 9.27, *SD* = 1.01).

Spearman’s correlations found Work Compatibility to be moderately correlated with both Emotional Compatibility (r_s_ = 0.50), and more strongly correlated with overall satisfaction (r_s_ = 0.72) and satisfaction with the match (r_s_ = 0.68). Emotional Compatibility was also correlated with satisfaction (r_s_ = 0.65) and match (r_s_ = 0.52). Social Compatibility (item 9) was correlated with Work Compatibility (r_s_ = 0.34) and with Emotional Compatibility (r_s_ = 0.32). The compatibility scales were correlated with the FES score as follows: Work Compatibility (r_s_ = 0.69), Emotional Compatibility (r_s_ = 0.60) and Social Compatibility (r_s_ = 0.42).

### 3.3. FES and Compatibility Differences According to Match/Mismatch Decision

The third goal was to see whether there were differences in FES and Compatibility scores between those who considered the handler/dog combination to be a matching success and those who considered it to be a mismatch (Table 6). Only 45 of the 50 handlers were able to indicate with certainty whether they considered the match to be a success (n = 37; 82.2%) or not (n = 8; 17.8%).

As shown in Table 6, the mean differences are greatest for the FES scores, the Work Compatibility scores, and satisfaction with the match and with the dog overall. The differences are less pronounced for the Emotional Compatibility item scores and for Social Compatibility. With respect to FES, the largest difference of 1.83 on the 4-point scale related to travel (mobility), followed by a difference of 1.68 for the dog’s behavior. The largest contrasts within the Work Compatibility items were observed for safety and control with mean differences of 5.24 and 3.78 respectively.

### 3.4. Assocations between Motivation to Acquire and Succeed with a Guide Dog and Fulfillment of Expectations, Compatibiity and Matching Success

To achieve the last goal, the motivation to acquire and to succeed with the dog was rated on a 10-point rating scale (with 1 being ‘not at all motivated’ and 10 being ‘extremely motivated’); ratings of motivation to acquire ranged from 1–10 with a mean of 7.88 (*SD* = 2.40) and ratings of motivation to succeed from 6–10 (*M* = 9.40, *SD* = 0.93).

Correlations between the motivation scores and the FES and compatibility subscales are presented in Table 7, showing that motivation to succeed with a dog was more strongly associated with the fulfillment of expectations and compatibility scores than was the motivation to acquire a dog. Work Compatibility was most strongly correlated with the motivation to succeed.

To see whether the motivation ratings were linked to perceptions of matching success, mean scores for the successful match and mismatch groups were compared. Again, the motivation to succeed appeared to have more discriminative power than the motivation to acquire a dog; mean scores for motivation to succeed were 9.49 for the successful match group and 8.88 for the mismatch group. Mean scores for motivation to acquire a dog were 7.89 for the successful match group and 7.25 for the mismatch group.

## 4. Discussion

The goals of this paper were: firstly, to see whether having a first guide dog met the expectations of people with a vision impairment; secondly, whether they felt that they were compatible with their assigned dog; thirdly, whether ratings of fulfillment of expectations and compatibility differed according to whether or not the participants considered the handler/guide dog pairing a success; and finally, to see how motivation to acquire and succeed with a dog was related to fulfillment of expectations, compatibility and perceptions of whether or not the pairing was a matching success.

The FES results suggest that nearly all the handlers’ expectations of guide dog use were at least met, and mostly exceeded, by their first dog. This supports earlier findings by Whitmarsh [5] that expectations and perceived benefits of guide dog use are well aligned. The example given by Whitmarsh (5) was that 90% of those indicating that improved mobility was a benefit of ownership, had applied for a dog with that reason in mind. Similarly, other studies [8,9] found that all participants had mobility-related expectations of their prospective guide dogs, expecting them to be a superior aid to a long cane, to make journeys safer and faster, and unfamiliar environments more accessible. The authors reported that these expectations were satisfied in the main. Doorish [22] also found that a desire to overcome the limitations of using a long cane was instrumental in the decision to apply for a guide dog. The way in which this expectation was met was described by his participants who defined the cane as an obstacle locator and the dog as an obstacle avoider, thus improving speed, confidence and decreasing pain from contact with obstacles when travelling. This equates to the travel component of the FES and also to the safety and mobility components of compatibility in the current study.

Lane and colleagues [23] touched on other expectations of guide dog use with their focus group participants who described a range of ambitions they had held prior to getting a dog that had been subsequently achieved. These included engaging in more exercise and having an enhanced sense of well-being. Overall, the authors concluded that handlers associated improved fitness, improved emotional and physical health, higher self-esteem and more social engagement with using a canine guide [23]. Companionship was also mentioned as an advantage of guide dog use. The greatest fulfillment in our study was associated with companionship, with only one participant indicating that their expectations on this front were only partially met.

Responses to the 10 compatibility questions were combined, based on their qualitative content, into three weak to moderately correlated components or subscales. The subscales constituted different aspects of the relationship between handler and dog related to work, emotional and social compatibility. Although the ratings of compatibility were very high overall, there was sufficient variation to demonstrate apparent mean differences between those who considered their pairing to be a matching success and those who did not. Compatibility between people and their animal companions has been explored before, a general measure having been developed in the context of cat and dog ownership [17], and a compatibility of activity preferences measure for dogs and their owners [13]. However, neither of these was appropriate for the current context, as guide dogs play such a specific and essential role with and for their handlers. A service dog matching tool was developed by Zapf and Rough [24], involving assessment, of the client only, around functional needs, prior experience with animals, typical activity level, affective state and resources. It was described in a recent doctoral thesis as the only published matching tool but one that is too brief and possibly excluding of factors that are important to a successful service dog/client match [25]. A profiling tool was recently developed by Meyer and colleagues [26] which can be used to classify people with impaired vision into four different types of travelers in order to match them with appropriate guide dogs. These ideas are based predominantly on travel needs and the questions asked only touched on more social aspects of guide dog use and care.

When looking at the differences in ratings of expectation fulfillment and the different types of compatibility for the well-matched and mismatched groups, it was evident that the largest differences were linked to the work-related aspects of the relationship. This highlights the importance of guide dogs being good at their job and compatible with the handler’s vision-related needs for the match to be classified as successful. A big difference in compatibility between the groups was related to satisfaction with the safety of the dog as a mobility aid. While it is outside the scope of this paper to discuss the nuances of guide dog training, this draws attention to the necessity of guide dog schools to focus on key skills such as straight line walking, indicating curbs/stairs/intersections by stopping, understand and respond to the handler’s verbal instructions and ignore distractions such as other dogs, cats and food. It appears that there was less variation in emotional responses to the dogs across the two groups, and it may be that handlers get attached to their guide dog and feel there is a strong emotional bond between dog and handler regardless of how they perform as a working team. It must be remembered that although the dog is primarily a mobility aid, and work safety is paramount, it is also an animal companion and consequently provides the handler with other social and emotional benefits.

It has been suggested that there are important aspects of guide dog behavior that are not prioritized in assessments. Craigon et al. [27] point out that the guide dog is only working for a small part of the day, with the rest of the time being spent just as a dog in the handler’s household. The need for compatibility applies to non-working time as well, including time a dog may spend, for example, lying under a desk in a handler’s office. As found in other studies by Lloyd and colleagues [8,15,20], York and Whiteside [28] observed that their focus group participants considered the way in which their guide dog behaved when out of harness was as important as when it was working, and suggested the need for better understanding of the prospective handler’s non-work as well as work expectations/requirements of a guide dog. As the emphasis on training a guide dog is predominantly on how the dog works in order to support the handler’s mobility, it would befit trainers/instructors to also focus on the social aspects (non-work) of the dog’s behavior and consider what the social expectations of the handler might be when making matching decisions. Puppy walkers (also known as puppy raisers or puppy fosters) will have a good sense of their previous charge’s social ‘at home’ behavior and could be asked to fill in any knowledge gaps to provide a fuller character profile of the dog, as recommended by York and Whiteside [28]. Gravrok et al. [14] explored challenges experienced by first-time handlers of a variety of assistance dogs and concluded that handlers’ medical conditions, cognitive ability and social environment (as well as dog-related factors) were important and should be considered prior to placing an assistance dog. This information may be helpful to the potential application of the findings of the present study for guide dog handlers with difficulties other than visual impairment.

When we looked at how motivated handlers rated themselves as being to firstly acquire a guide dog and then succeed with the dog they were matched with, it was clear that the motivation to succeed had stronger connections with the fulfillment of expectations, compatibility and the perceived success of the match. Perhaps motivation to succeed reflects the idea of readiness for change, as encompassed by the Transtheoretical Model of Health Behavior Change (also known as the Transtheoretical Model) [29]. The Transtheoretical Model assesses individuals’ readiness to progress through various stages of change in six steps. These are: precontemplation, contemplation, preparation, action, maintenance, and termination. Making the decision to get a guide dog represents a huge change in one’s life, with both positive and negative implications, and it may be that people who are more prepared to be flexible and modify certain attitudes and/or behaviors are more likely to make the new relationship work.

### Limitations of the Study

Characteristics of well-matched versus mismatched dogs was explored by Lloyd et al. [15] where handlers were asked to comment on what was good and bad behaviorally (and physically) about their dogs (118 handler-dog pairings). The most reported good behavior concerned social behavior including the dog being personable and well behaved at home and in other social settings, followed by the dog’s capacity to work and guiding ability. The bad behaviors most reported concerned work, closely followed by poor social behaviors. A limitation of the current study was that the only social aspect we enquired about was the *effect* of the dog on social interaction rather than its social or non-working behavior. However, some of this may have come into participants’ ratings of their guide dog as a companion, as poor social behavior could be assumed to have a negative impact on companionship. Other limitations of this study include the relatively small sample size, although it should be acknowledged that it represents over a fifth of all current and previous guide dog users in New Zealand at the time. The relatively broad scope of the questions could also perhaps be viewed as a limitation. This was the first study to examine this topic, and we wished to take a wide exploratory approach, but had we divided the compatibility and expectations items into more precisely worded questions we could have gleaned more information about more specific aspects of these constructs. It is recommended that this be explored further in future research.

It is interesting to note that only a small fraction (<1–2%) of people in NZ and in other countries [30] who are eligible to apply for a guide dog (i.e., are legally blind) use or are waiting for a dog. There are many reasons why this might be; secondary disabilities such as diabetes, hearing loss and neurological conditions may not necessarily preclude eligibility [21], but it could mean that those who have or want a dog are already highly motivated compared to the blind and low vision community.

It is generally believed that applicants need to be able to demonstrate the need for a dog as a mobility aid to help them travel safely and with confidence. In NZ it is not mandatory for people to have received formal orientation and mobility (O&M) training prior to applying, but it is considered useful if people are confident in their travel ability. For some people, having poor or no long cane mobility skills may not be detrimental to travelling with a dog. A study by Lloyd and colleagues [4] showed that peoples’ self-professed degree of O&M skills before they got a dog did not affect the level of their perceived travel performance. Indeed, those who described themselves as poor travelers appeared to gain the most from using a guide dog—provided the match was considered successful, and applicants were well oriented to their usual destinations. This study [4] indicated that guide dog use also alleviated mobility restrictions for people with non-visual conditions such as repetitive strain injuries caused by long cane use, and hearing loss—the latter of which can impede orientation and is of greater concern with the current manufacturing of quieter cars. These findings support Milligan’s [31] suggestions regarding who may benefit from using a guide dog and should be useful for instructors when assessing applications for guide dogs.

Having a guide dog is a big commitment and not everyone who is eligible chooses to, or should, have one. As with any assistance dog or pet dog, responsible ownership requires a commitment to provide for all the requirements of the dog—food, exercise, housing, reward-based training, love and affection, grooming and veterinary care [32]. It is also necessary for users to learn about how dogs communicate and learn, and to thoroughly research the basics of dog care before acquiring the dog to ensure the physiological, behavioral and social needs of the dog are met [32]. The onus need not be on the applicant to prove why they are eligible for a dog, but on the guide dog schools to show why certain individuals might not be trained to work with a dog. Guide dogs can make profound differences in the lives of those who use them, and awareness should be raised around these issues.

## 5. Conclusions

The findings of this study of 50 first time guide dog handlers demonstrate firstly that the dogs are at least meeting and generally exceeding their expectations. The high average ratings of compatibility were notable, particularly with respect to the emotional compatibility between handler and dog. Comparing responses of those who felt the handler-dog pairing was a good match with those who felt it was a mismatch revealed that it was the working aspects of the relationship that differentiated the two groups. These aspects specifically related to travel, the dog’s behavior, safety while travelling, and ability to control the dog—all important parts of a working relationship which must be prioritized when training the guide dog team. However, the sheer number of aspects of life with a guide dog, beyond the complexities of the working relationship, suggest that a more nuanced compatibility measure accommodating both positive and negative aspects of the relationship would be beneficial. Such a tool could assist with initial matching and could also be used during training and follow-up to assess how well the match is working out and identify areas where changes could be made to enhance the likelihood of overall fulfillment and success.

## Figures and Tables

**Table 1:** Compatibility item content and associated variable name.

Item Content	Variable Name
The emotional connection (bond) between handler and dog (two-way relationship)	Bond
Attachment of the handler to the dog (one-way relationship)	Attachment
The handler’s ability to control the dog	Control
Satisfaction with the dog as a mobility aid	Mobility
Satisfaction with the safety of the dog as a mobility aid	Safety
Overall satisfaction with the dog	Satisfaction
Overall success of the match	Match
Overall compatibility of the pairing	Compatibility
Satisfaction regarding the dog’s effect on social interactions	Social
Satisfaction with the dog as a companion	Companionship

**Table 2:** How well expectations of guide dog use were met by first guide dogs, with chi-square goodness of fit values.

	Expectations (%)				
Item	Not Met (1)	Somewhat Met (2)	Met (3)	Exceeded (4)	χ^2^
Travel	8.0	10.0	14.0	68.0	51.65 **
Social function	2.0	2.0	36.0	60.0	56.60 **
Companionship	0.0	2.0	30.0	68.0	70.26 **
Dog’s behavior	8.0	20.0	28.0	44.0	13.68 *
Dog’s health	4.0	16.0	34.0	46.0	20.88 **

* *p* < 0.01, ** *p* < 0.001.

**Table 3:** Spearman’s correlations between the fulfilment of expectations items.

Item	2	3	4	5
1. Travel	0.45	0.28	0.63	0.32
2. Social function	.	0.39	0.30	0.33
3. Companionship		.	0.36	0.36
4. Dog’s behavior			.	0.33
5. Dog’s health				.

**Table 4:** Compatibility item medians and ranges.

Item	Median	Range
Bond	10.0	5–10
Attachment	10.0	7–10
Control	8.5	1–10
Mobility	10.0	2–10
Safety	9.0	1–10
Satisfaction	10.0	3–10
Match	10.0	2–10
Compatibility	10.0	4–10
Social	9.5	6–10
Companionship	10.0	5–10

**Table 5:** Spearman’s correlations between the compatibility items.

Items	2	3	4	5	6	7	8	9	10
1. Bond	0.80	0.30	0.39	0.37	0.62	0.51	0.59	0.25	0.77
2. Attachment	.	0.27	0.38	0.30	0.54	0.40	0.54	0.36	0.75
3. Control		.	0.63	0.57	0.52	0.54	0.32	0.30	0.19
4. Mobility			.	0.71	0.63	0.58	0.54	0.24	0.38
5. Safety				.	0.72	0.69	0.52	0.41	0.28
6. Satisfaction					.	0.87	0.67	0.38	0.55
7. Match						.	0.58	0.46	0.43
8. Compatibility							.	0.23	0.44
9. Social								.	0.31
10. Companionship									.

**Table 6:** A comparison of fulfilment of expectations (FES) and compatibility mean scores for the successfully matched (Match) (n = 37) and mismatched (Mismatch) (n = 8) groups with scale scores in bold type.

FES	Match	Mismatch	Compatibility	Match	Mismatch
Travel	3.83	2.00	Safety	9.24	4.00
Social function	3.87	3.50	Mobility	9.46	5.88
Companionship	3.85	3.83	Control	8.78	5.00
Dog’s behavior	3.43	1.75	Compatibility	9.54	7.63
Dog’s health	3.35	3.13	**Work Compatibility**	**9.26**	**5.63**
**FES**	**3.69**	**2.97**			
			Attachment	9.57	9.25
			Companionship	9.38	9.00
			Bond	9.35	8.35
			**Emotional Compatibility**	**9.43**	**8.88**
			**Social Compatibility**	**8.97**	**8.38**
			Match	9.54	4.25
			Satisfaction	9.51	5.88

**Table 7:** Spearman’s correlations between ratings of motivation to acquire and succeed with a dog and the FES and Compatibility subscales (Work Compatibility, Emotional Compatibility and Social Compatibility) scores.

Item	Motivation to Acquire	Motivation to Succeed
FES	0.04	0.31
Work Compatibility	0.17	0.50
Emotional Compatibility	0.21	0.28
Social Compatibility	0.04	0.42

## Data Availability

The datasets for this article are not publicly or otherwise available due to issues of confidentiality.
